# American Medical Association

**Published:** 1877-06-20

**Authors:** 


### AMERICAN MEDICAL ASSOCIATION.

The American Medical Association assembled in Chicago on the 7th inst. The meeting was of full average attendance, there being present six hundred and fifty members. Many new members were initiated. President Bowditch called the Convention to order, and addressed the body. The Secretary read various reports from different States.

The Committee on Publication rehearsed the many difficulties they had encountered with the printers, &c., as apologetic for the late appearance of the transactions of last meeting.

The Treasurer's Report showed the Treasury exausted, by reason of $6,000 paid for Prize Essay, in addition to the outlay for Transactions.

The address of Dr. E. M. Hunt, on Public Hygiene, expressed the hope, as not unreasonable, that we will yet discover means of protecting the system against zymotic diseases.

The Librarian's Report shows the Association possessed of 2,034 volums.

Dr. Squibb read a paper on the Revision of the U. S. Pharmacopoea, and favored the appointment of a committee to report at next meeting. He was opposed by Prof. Wood, in a speech of some vehemence. The whole matter was, finally, postponed indeffinitely.

Meetings were held by the several Sections on Obstestrics, Surgery, Anatomy, &c., wherein many important matters were discussed. Reports are yet too meager for the publication of the details of the subject matter of the proceedings. These we hope to obtain hereafter for the benefit of our readers.

The Committee on Nominations, report the following as officers for the ensuing year, to wit: For President, T. G. Richardson, of Louisiana; Vice-Presidents, J. P. White, N. Y.; M. Gunn, Illinois; G. W. Russel, Connecticut, and A. Dunlop,

Section of Medicine, Materia Medica and Physiology: Chairman, A. L. Loomis, N. Y.; Secretary J. H. Ethridge, Illinois.

Section of Obstetrics and Disease of Women and Children: Chairman, E. W. Jenks, Michigan; Secretary, H. O. Marcy, Mass.

Section of Surgery and Anatomy: Chairman H. N. Smith, of Pennsylvania; Secretary, E. J. Easly, Arkansas.

Section of Medical Jurisprudence: Chairman, W. Kempster, of Miss.; Secretary, E. A. Hildreth, West Virginia.

Section of State Medicine and Hygiene: Chairman, J. L. Cabell, of Va.; Secretary, Z. E. Marsh, N. J.

Time and place of next meeting—Buffalo, N. Y., first Tuesday of June, 1878.

